# Parkinson's Disease and Respiratory Failure: Epidemiologic Insights of Two Decades From the CDC‐WONDER Database

**DOI:** 10.1002/brb3.71756

**Published:** 2026-09-23

**Authors:** Maryam Saghir, Amna Amir Jalal, Eshal Saghir, Kashmala Rahl, Nida Shoaib, Muhammad Affan, Mahtab Zafar, Nara Miriam Michaelson, Kamil Ahmad Kamil

**Affiliations:** ^1^ M.B.B.S Jinnah Sindh Medical University Karachi Pakistan; ^2^ M.B.B.S Dow University of Health Sciences Karachi Pakistan; ^3^ M.B.B.S Rahbar Medical and Dental College Lahore Pakistan; ^4^ Beth Israel Deaconess Medical Center, Harvard Medical School Boston Massachusetts USA; ^5^ Chief, Internal Medicine Department Nasrat Bashir Curative Hospital Kandahar Afghanistan

**Keywords:** CDC WONDER, epidemiology, older adults, Parkinson's disease, respiratory failure

## Abstract

**Introduction:**

Parkinson's disease (PD) is the second most common neurodegenerative disorder, with increasing prevalence and mortality among older adults. Beyond motor dysfunction, PD contributes to respiratory impairment through muscle weakness, and airway obstruction. Respiratory failure, a major global health burden, is increasingly recognized as a significant cause of fatality in advanced PD.

**Methods:**

We analyzed PD (ICD‐10 code: G20) and respiratory failure (ICD‐10 code: J96)‐related mortality in older adults, using the CDC‐WONDER database from 1999 to 2020. Age‐Adjusted Mortality Rates (AAMR) per 100,000 were calculated and categorized by demographics and region. Joinpoint regression was used to estimate Annual Percent Change and Average Annual Percent Change (AAPC).

**Results:**

A total of 59,168 PD and respiratory failure‐related deaths were recorded among older adults aged ≥65 years. The AAMR observed a significantly steep incline from 5.5 in 1999 to 11.38 in 2020 (AAPC: 3.10%; 95% CI: 2.01 to 4.20, *p* < 0.01). Men demonstrated higher overall AAMR than women. Among different races and ethnicities, Non‐Hispanic White population had the highest overall AAMR. Regionally, the highest overall AAMR was recorded in the West and Metropolitan areas.

**Conclusion:**

This study highlights a marked rise in PD and respiratory failure‐related deaths in the United States, with notable disparities across demographics and regions. Mortality was higher in males and non‐Hispanic Whites, with the West and urban areas showing sharp increases, reflecting limited access to special care. These findings underscore widening inequities and the need for targeted policies, equitable resource allocation, and further research on underlying social and healthcare determinants.

## Graphical abstract

Mortality from Parkinson's disease with respiratory failure more than doubled among US adults aged ≥65 years from 1999 to 2020, with disproportionately higher burdens among men and non‐Hispanic White individuals. Rural areas experienced a particularly steep increase in the late period, highlighting significant geographic disparities in outcomes and access to specialized care.

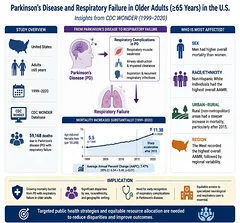

## 1 Introduction

Parkinson's disease (PD), the second most common progressive neurodegenerative disease after Alzheimer's disease (AD) is primarily characterized by motor symptoms that significantly affect a person's quality of life (Luo et al. 2025). Currently, about 1 million people in the United States have PD (Spenser), with an estimated 60,000 new cases diagnosed each year. Retrospective studies of people with PD aged 65 ≥ showed a total of 831,793 deaths between 1999 and 2020 (Tharwani et al. 2024). Concurrently, a separate investigation revealed an increase in the age‐adjusted mortality rate (AAMR), rising from 5.4 per 100,000 in 1999 to 8.8 per 100,000 by 2019 (Rong et al. 2021). The World Health Organization (WHO) estimated that, in 2019, 8.5 million people globally were living with PD resulting in 329,000 deaths (World Health Organization 2023). The Global Burden of Disease Study (GBD) reported a 21.7% increase in the age‐standardized prevalence of Parkinson's disease in 2016, affecting 6.1 million people globally, with environmental exposures, particularly in high‐risk occupations, identified as key risk factors (GBD 2016 Parkinson's Disease Collaborators 2018; Tysnes and Storstein 2017).

Respiratory failure is a serious illness, characterized by the lungs' failure to maintain adequate gas exchange, which significantly impacts global mortality rates (Kempker et al. 2020). Between 2002 and 2017, the national adult incidence of respiratory failure strikingly increased by 197%, going from 429 to 1275 cases per 100,000 adults (GBD Chronic Respiratory Disease Collaborators 2020). Respiratory failure may present in either acute or chronic forms. The number of people with chronic respiratory diseases has grown dramatically around the world, with 544.9 million people affected in 2017, or 7.1% of the population. This is a big increase from estimates made in 1990 (GBD Chronic Respiratory Disease Collaborators 2020). With around 4 million premature deaths each year caused by chronic respiratory failure, mortality remains high; acute respiratory failure accounts for 2.6 million more fatalities globally. With age‐adjusted mortality rates rising from 74.9 per 100,000 in 2014 to 85.6 per 100,000 in 2018 (Parcha et al. 2021), a recent study reported 1,434,349 acute respiratory failure‐related deaths in the United States.

Parkinson's disease is characterized by progressive loss of dopaminergic neurons in the substantia nigra pars compacta and accumulation of misfolded α‐synuclein within Lewy bodies, leading to neuronal dysfunction and neurotoxicity (Wei 2022). Degeneration of basal ganglia motor circuits causes motor symptoms such as tremor, rigidity, bradykinesia, postural instability, and respiratory muscle weakness (Macphee and Stewart 2012), while involvement of other brain regions contributes to non‐motor symptoms including sleep disturbances, cognitive decline, and depression. Respiratory dysfunction in Parkinson's disease is multifactorial, involving respiratory muscle weakness, upper airway obstruction, impaired chest wall mechanics, and abnormal ventilatory control, ultimately contributing to respiratory failure and increased morbidity and mortality (Marini J. J. 2018; McMahon et al. 2023; Torsney and Forsyth 2017).

PD is predicted to become the next major source of mortality and morbidity. Though its influence is growing, there is little thorough information on its epidemiology and economic cost, stressing the need for more research. Public health planning, resource distribution, and the creation of targeted therapies need epidemiology and trends of PD. This review seeks to address that gap by analyzing PD and respiratory‐related mortality in the United States between 1999 and 2020. In addition, it examines racial disparities in comorbidity and mortality, as well as temporal trends and variations by sex, geographic region, and urban–rural residence, using data from the Centers for Disease Control and Prevention Wide‐Ranging Online Data for Epidemiologic Research (CDC‐WONDER) database.

## 2 Methods

### 2.1 Study Design

We conducted a retrospective analysis using death certificate data retrieved from the Centers for Disease Control and Prevention Wide‐Ranging Online Data for Epidemiologic Research (CDC WONDER) database and analyzed data for older adults aged 65 and older between 1999 and 2020 to assess long‐term temporal trends in mortality related to PD and respiratory failure, using the International Statistical Classification of Diseases and Related Health Problems—10th Revision (ICD‐10) as follows: G20 for PD and J96 for respiratory failure, both as multiple or contributing causes of death. These ICD codes have been previously used to identify PD and respiratory failure individually (Alam et al. 2026; Søgaard et al. 2016). In order to facilitate comparability of results across analyses, the research timeframe and overall methodological approach were chosen to be consistent with previously published CDC WONDER‐based mortality studies (Kumar et al. 2026; Saghir et al. 2026; Sohail et al. 2025). Institutional review board approval was not required for this study, as it used de‐identified public use data provided by the government and adheres to the Strengthening the Reporting of Observational Studies in Epidemiology (STROBE) guidelines for reporting (Von Elm et al. 2008).

### 2.2 Data Abstraction

This study categorized mortality data based on population size and demographic variables, including sex, age, race/ethnicity, region, and states. Place of death was classified into medical facilities, hospice, home, and nursing home/long‐term care facilities. Racial and ethnic categories were consistent with CDC WONDER classification and included non‐Hispanic (NH) White, NH Black or African American, Hispanic or Latino, NH American Indian or Alaskan Native, and NH Asian or Pacific Islander. The National Center for Health Statistics Urban–Rural Classification Scheme was used to assess the population by urban (where large metropolitan areas had populations ≥1 million, medium/small metropolitan areas had populations of 50,000–999,999, and rural populations were <50,000) counties per the 2013 US census classification (Aggarwal et al. 2021). Regions were evaluated according to the US Census Bureau definitions and included: Northeast, Midwest, South, and West (Ingram and Franco 2014).

### 2.3 Statistical Analysis

The outcome measures computed for analysis included crude and age‐adjusted mortality rates (CMRs and AAMRs) per 100,000 population from 1999 to 2020. AAMRs were determined by the direct method, standardized to the 2000 US standard population to enable comparisons across demographic and geographic groups (Anderson and Rosenberg 1998). CMRs were calculated by dividing the number of patients with PD and respiratory failure by the corresponding US population of that year. Joinpoint Regression Program (Joinpoint V 5.4.0.0, National Cancer Institute) was used to assess changes in PD and respiratory failure‐related mortality rates over time. Annual percent change (APC) and its associated 95% confidence intervals (CIs) in AAMR were determined using the Monte Carlo Permutation test (Joinpoint trend analysis software 2025). This method allows identification of significant changes in AAMR over time by fitting log‐linear regression models where temporal variation occurred. To indicate the overall trend in mortality from 1999 to 2020, average annual percentage change (AAPC) was calculated as a weighted mean of APCs, with associated 95% CIs. APCs were considered increasing or decreasing if the slope representing the change in mortality was significantly different from zero using two‐tailed *t* testing. A *p* value of less than 0.05 indicated statistical significance.

## 3 Results

Between 1999 and 2020, a total of 59,168 deaths related to PD and respiratory failure were recorded among adults aged 65 years and above in the United States. For the years 1999 to 2020, data on the place of death were available for 57,039 cases. The majority of these deaths occurred in Medical Facility (46.13%), followed by Nursing home (30.97%), Decedent's home (17.23%), and hospice setting (5.65%).

### 3.1 Annual Trends for PD and Respiratory Failure‐Related AAMR

**FIGURE 1:**
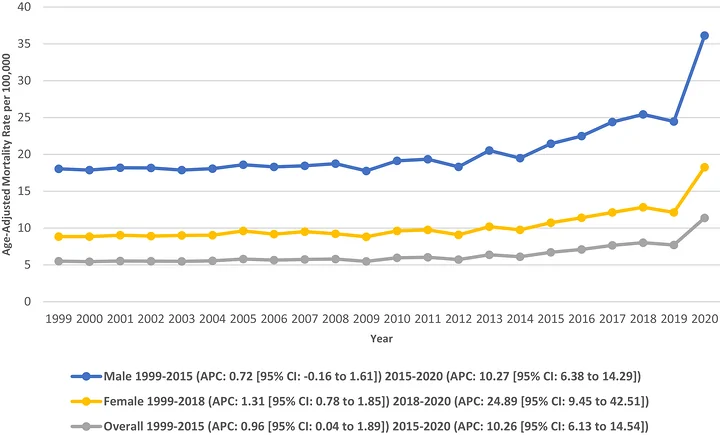
Overall and sex‐stratified Parkinson's disease and respiratory failure‐related AAMRs per 100,000 in older adults in the United States 1999–2020.

The overall AAMR for PD and respiratory failure‐related deaths among older adults changed from 5.5 in 1999 to 11.38 in 2020, with an AAPC of 3.10 (95% CI: 2.016 to 4.20, *p* < 0.01). Notably, the AAMR experienced a significant increase from 5.5 in 1999 to 6.71 in 2015 (APC: 0.96; 95% CI: 0.04 to 1.89, *p* = 0.04), followed by a significant increase from 6.71 in 2015 to 11.38 in 2020 (APC: 10.26; 95% CI: 6.13 to 14.54, *p* < 0.01) (Figure 1; Tables S1 and S3).

### 3.2 PD and Respiratory Failure‐Related AAMR Stratified by Sex

Throughout the study period, men exhibited substantially higher AAMRs than women (overall AAMR for men: 10.52, 95% CI: 10.41 to 10.63, for women: 3.95, 95% CI: 3.9 to 4.01). The AAMR for men increased from 1999 to 2020 [men: AAPC: 2.91 (CI: 1.88 to 3.96, *p* < 0.01)]; while for women, it increased between 1999 and 2020 [women: AAPC: 3.35 (CI: 2.07 to 4.65, *p* < 0.01)].

The AAMR for men demonstrated a non‐significant increase from 9.2 in 1999 to 10.73 in 2015 (APC: 0.72; 95% CI: –0.16 to 1.61, *p* = 0.1). Then, the AAMR significantly increased from 10.73 in 2015 to 17.86 in 2020 (APC: 10.27; 95% CI: 6.38 to 14.29, *p* < 0.01). The AAMR for women experienced a significant increase from 3.3 in 1999 to 4.8 in 2018 (APC: 1.31; 95% CI: 0.78 to 1.85, *p* < 0.01), followed by a significant increase from 4.8 in 2018 to 6.88 in 2020 (APC: 24.89; 95% CI: 9.45 to 42.51, *p* < 0.01) (Figure 1; Table S4).

### 3.3 PD and Respiratory Failure‐Related AAMR Stratified by Race/Ethnicity

Significant variability in the number of deaths was found among different racial/ethnic groups, with the highest number of deaths occurring in NH White individuals, followed by Hispanic individuals, NH Black individuals, and lowest in NH Asian or Pacific Islanders. AAMRs were highest among NH Whites, followed by Hispanic or Latinos, NH Asian or Pacific Islander, and NH Black populations (overall AAMR: NH White: 6.81, Hispanic or Latino: 6.6, NH Asian: 6.31, NH Black or African American: 3.52).

**FIGURE 2:**
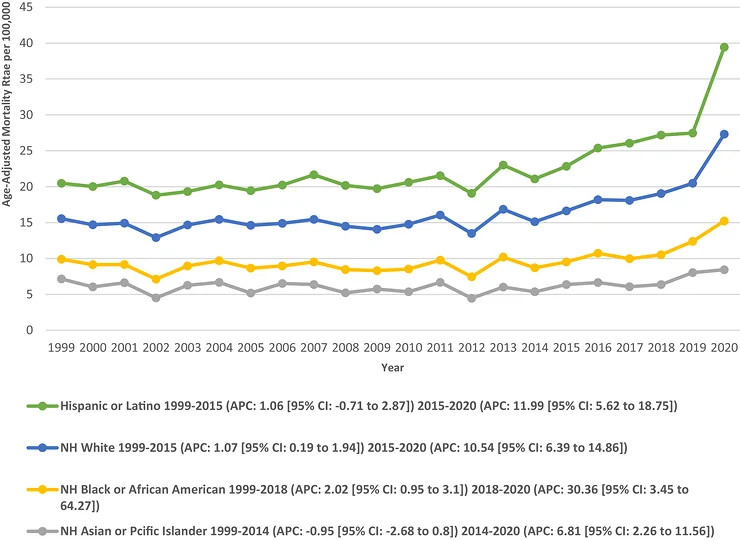
Parkinson's disease and respiratory failure‐related AAMRs per 100,000 stratified by race in older adults in the United States 1999–2020.

The AAMR of all the races changed to variable degrees from 1999 till 2020, with the non‐significant increase in NH Asians (AAPC: 1.20, 95% CI: –0.44 to 2.87, *p* = 0.15), followed by significant increase in NH Whites (AAPC: 3.25, 95% CI: 2.17 to 4.33, *p* < 0.01), Hispanic (AAPC: 3.56, 95% CI: 1.71 to 5.45, *p* < 0.01), followed by significant increase in NH Black individuals (AAPC: 4.43, 95% CI: 2.13 to 6.75, *p* < 0.01) (Figure 2; Table S5).

### 3.4 PD and Respiratory Failure‐Related AAMR Stratified by Census Regions and States

Throughout the study timeframe, between 1999 and 2020, the greatest mortality was exhibited by the South, followed by the West, the Northeast, and the Midwest. The study concluded that the Northeast region had the greatest AAPC (4; 95% CI: 2.82 to 5.19; *p* < 0.01), followed by the Midwest (AAPC: 3.88; 95% CI: 2.44 to 5.34; *p* < 0.01), South (AAPC: 3.2; 95% CI: 1.99 to 4.43; *p* < 0.01), and finally the West (AAPC: 1.9; 95% CI: 0.89 to 2.92; *p* < 0.01).

**FIGURE 3:**
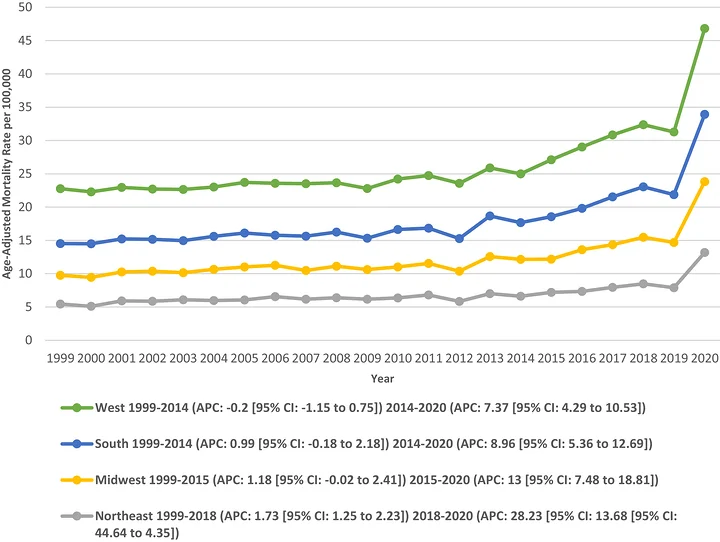
Parkinson's disease and respiratory failure ‐related AAMRs per 100,000 stratified by census region in older adults in the United States 1999–2020.

The South demonstrated a non‐significant change in AAMR from 4.78 in 1999 to 5.51 in 2014 (APC: 0.99; 95% CI: –0.18 to 2.18; *p* = 0.09), followed by a significant increase in AAMR from 5.51 in 2014 to 10.1 in 2020 (APC: 8.96; 95% CI: 5.36 to 12.69; *p* < 0.01). The AAMR showed variation in the West region, beginning from 8.24 in 1999 to 7.32 in 2014 (APC: –0.2; 95% CI: –1.15 to 0.75; *p* = 0.66). This was followed by a significant increase from 7.32 in 2014 to 12.9 in 2020 (APC: 7.3; 95% CI: 4.29 to 10.53; *p* < 0.01). Data analyzed for the Northeast showed an initial significant increase in AAMR from 5.44 in 1999 to 8.47 in 2018 (APC: 1.73; 95% CI: 1.25 to 2.23; *p* < 0.01) and then a significant increase from 8.47 in 2018 to 13.18 in 2020 (APC: 28.23%; 95% CI: 13.68 to 44.64; *p* < 0.01). AAMR in the Midwest saw a non‐significant change from 4.31 in 1999 to 5 in 2015 (APC: 1.18; 95% CI: –0.02 to 2.48; *p* = 0.05), followed by a significant increase from 5 in 2015 to 10.64 in 2020 (APC: 13; 95% CI: 7.48 to 18.81; *p* < 0.01) (Figure 3; Table S7).

**FIGURE 4:**
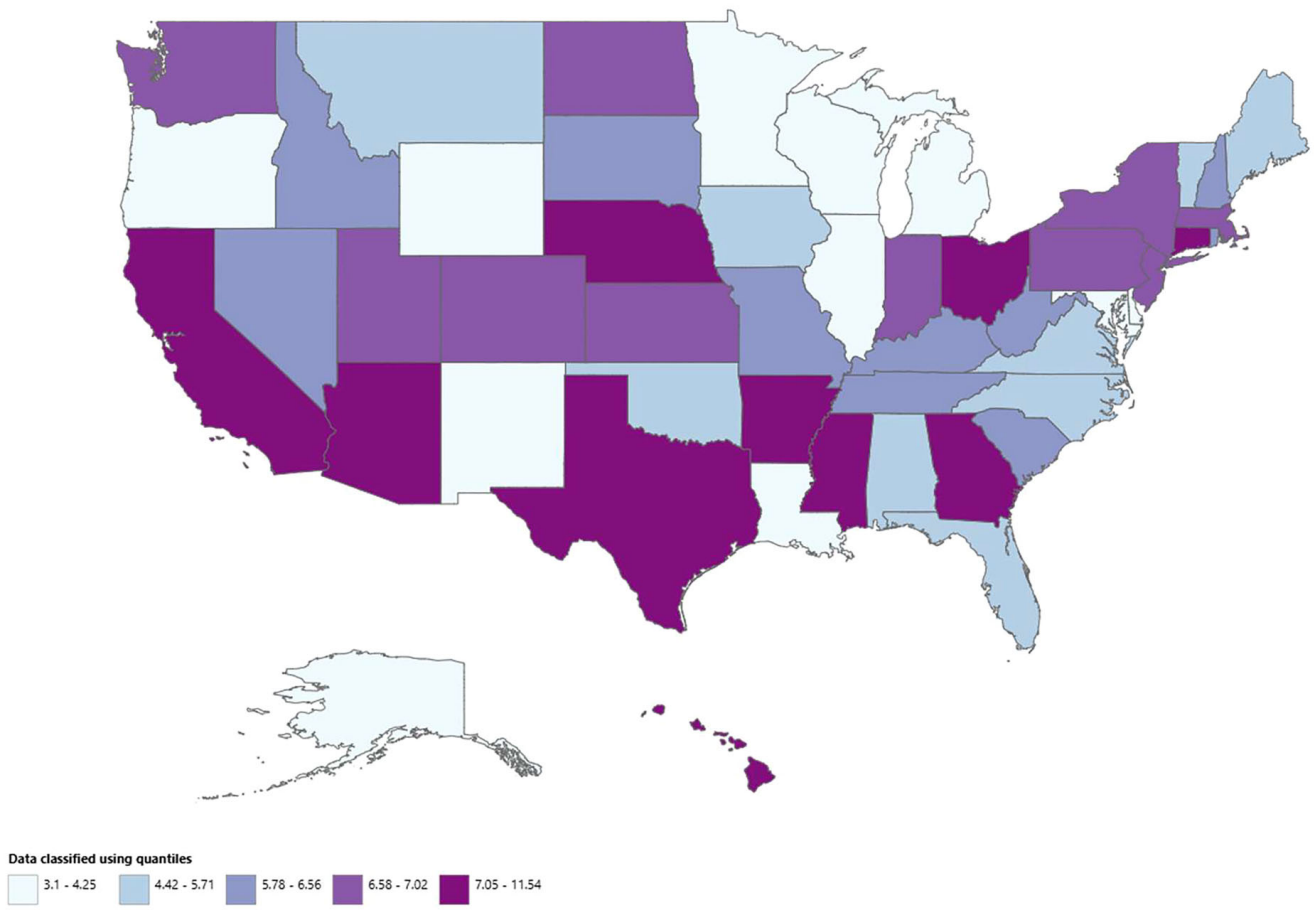
PD and respiratory failure‐related AAMRs per 100,000 stratified by states in the United States, 1999–2020.

Between 1999 and 2020, states that were classified within the top 90th percentile had nearly 3 times the AAMRs compared with states that were classified under the lower 10th percentile. Hence, states that consistently made it into the top 90th percentile were Nebraska (11.54), California (10.57), Connecticut (10.1), Mississippi (9.38), and Arkansas (9.13) (Figure 4; Table S6).

### 3.5 PD and Respiratory Failure‐Related AAMR Stratified by Urbanization

**FIGURE 5:**
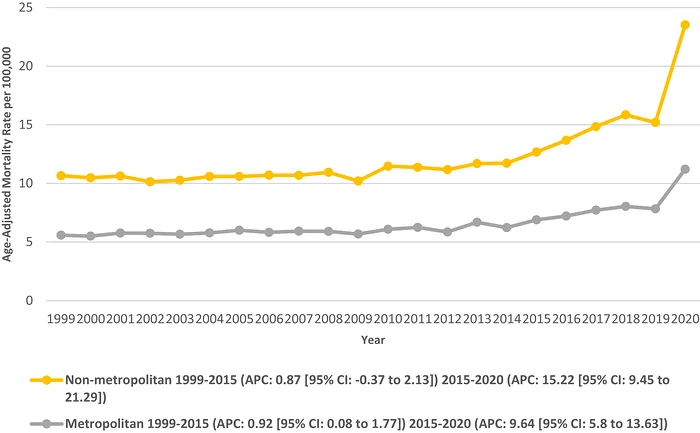
Parkinson's disease and respiratory failure ‐related AAMRs per 100,000 stratified by urban–rural classification in older adults in the United States 1999–2020.

**FIGURE 6:**
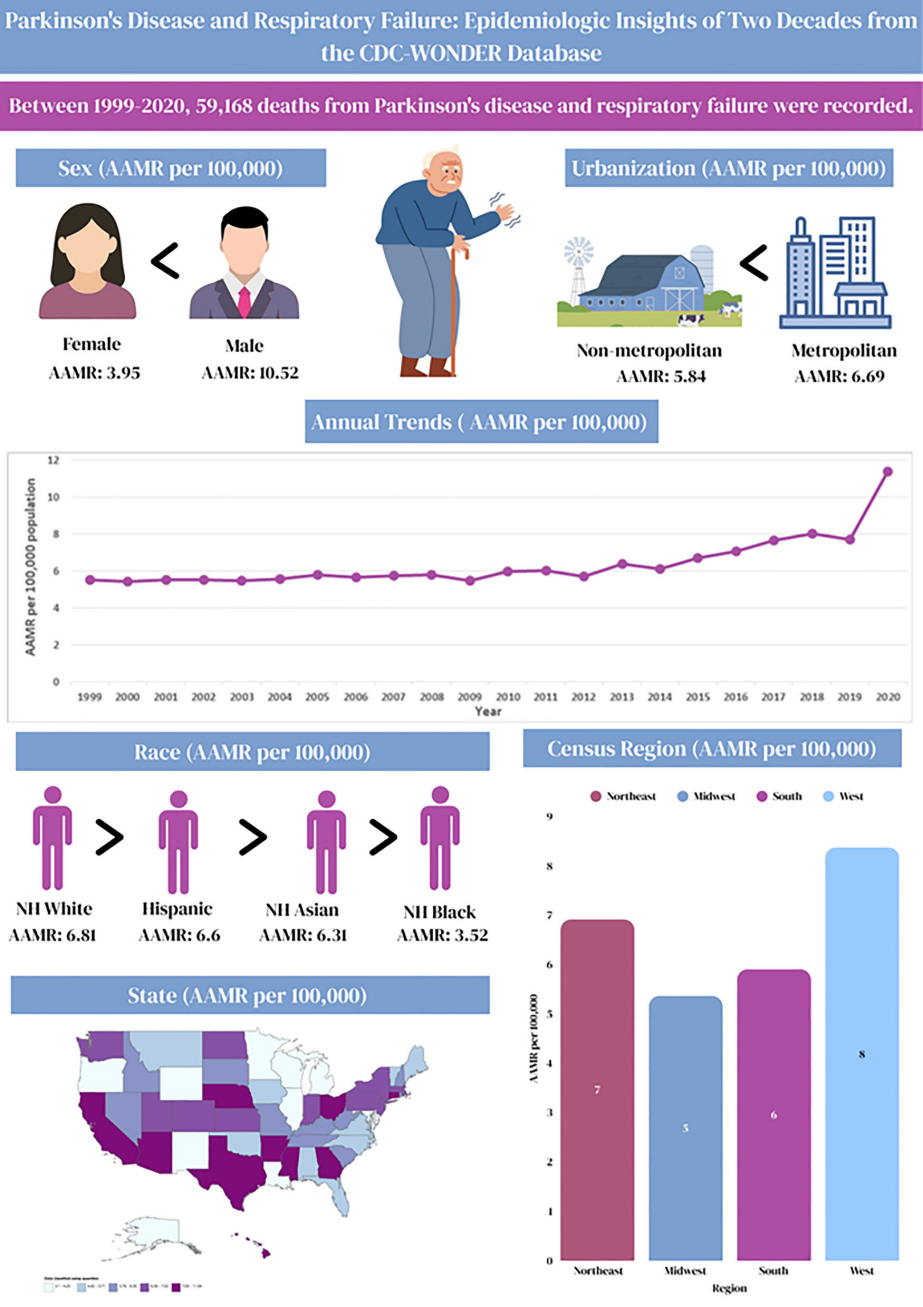
**Summary of our significant findings is shown in this central illustration**.

Between 1999 and 2020, the majority of deaths occurred in metropolitan areas as compared to non‐metropolitan areas. A relatively higher AAPC was observed in non‐metropolitan areas (4.11; 95% CI: 2.63 to 5.62; *p* < 0.01), whereas metropolitan areas had an AAPC value of 2.93 (95% CI: 1.92 to 3.96; *p* < 0.01). From 1999 to 2020, non‐metropolitan areas had an overall lower AAMR than metropolitan areas, with overall AAMRs of 5.84 and 6.69, respectively. Non‐metropolitan areas showed a non‐significant change in AAMR from 5.08 in 1999 to 5.79 in 2015 (APC: 0.87; 95% CI: –0.37 to 2.13; *p* = 0.16) and then a significant increase from 5.79 in 2015 to 12.3 in 2020 (APC: 15.22; 95% CI: 9.45 to 21.29; *p* < 0.01). Metropolitan areas observed a significant increase in AAMRs from 5.58 in 1999 to 6.89 in 2015 (APC: 0.92; 95% CI: 0.08 to 1.77; *p* = 0.03) and then a significant increase from 6.89 in 2015 to 11.21 in 2020 (APC: 9.64; 95% CI: –5.80 to 13.63; *p* < 0.01) (Figures 5 and 6; Table S7).

## 4 Discussion

The present investigation revealed a notable upward trajectory in mortality rates stemming from PD complicated by respiratory failure within the elderly population of the United States from 1999 to 2020. A pronounced escalation in AAMR was evident post‐2015, with males consistently demonstrating a significantly greater mortality burden compared to females. In terms of racial and ethnic demographics, White individuals represented the largest proportion of fatalities, although Hispanic and Black populations exhibited considerable increases over the observed periods. Geographically, the South and West reported the highest absolute mortality, while the Northeast and Midwest demonstrated the steepest upward trends. Although non‐metropolitan regions had lower overall AAMRs than metropolitan areas, they showed a faster rate of increase during the latter half of the study period.

Several interrelated factors contribute to the pathophysiology of respiratory failure in PD, which may explain the rise in PD and respiratory failure–related mortality observed in this study. First, the incidence and prevalence of PD rise markedly with age, which means that an aging population naturally increases the pool of individuals at risk for advanced disease and its complications (Docu Axelerad et al. 2021; Murakami et al. 2023). Therapeutic developments in PD treatment, including deep brain stimulation and dopaminergic medicines, have also enhanced motor symptom control and lengthened patient lifespan (Hitti et al. 2019; Lindvall et al. 1994). This elevates the probability of late‐stage problems like respiratory failure, thereby contributing toward increased mortality observed in this population (D'Arrigo et al. 2020; Won et al. 2021). Third, PD hampers respiratory function by stiffening the chest muscles and bradykinesia of the respiratory muscles. Dysfunction of the muscles controlling the throat and chest reduces the cough reflex, hence preventing proper airway closure during swallowing, ultimately enabling bacteria‐laden fluids to enter the lung cavity. This markedly increases the risk of aspiration and recurrent pneumonia, both of which are major contributors to respiratory compromise and can ultimately escalate to fatal outcomes in patients with PD (Kaczyńska et al. 2022). Finally, individuals with PD frequently carry additional age‐related comorbidities, such as cardiovascular disease or chronic obstructive pulmonary disease, and exhibit increasing frailty. These conditions synergistically compromise respiratory resilience, making them more prone to decompensation under stress and respiratory failure (Santiago et al. 2017).

Additionally, mortality rates increased both in men and women; however, we observed higher AAMRs in men during this timeframe. This trend can be explained by the fact that men bear a higher lifetime risk of getting Parkinson's than women, and in the long run, this increases the absolute number of men who are at risk for respiratory complications (GBD 2016 Parkinson's Disease Collaborators 2018; Zirra et al. 2022). According to Cattaneo and Pagonabarraga (2025), the prevalence of PD is 1.5 times higher in men than in women. Sex‐specific respiratory vulnerabilities also enhance this difference. Men tend to manifest impaired ventilatory efficiency, compromised respiratory muscle endurance, and increased incidences of cardiopulmonary comorbidities like chronic obstructive pulmonary disease, which can worsen respiratory failure when superimposed on PD‐related dysfunction (Aryal et al. 2014; LoMauro and Aliverti 2018). In women, estrogen is believed to exert neuroprotective effects through antioxidative and anti‐inflammatory mechanisms that counteract neuronal degeneration and slow the progression of PD (Vegeto et al. 2008). Also, in women, the development of symptomatic PD may be delayed by higher physiological striatal dopamine levels, possibly due to the activity of estrogens. This may account for epidemiological findings of lower frequency and older age of onset in women. Additionally, a 2007 study demonstrated that women are more likely to develop tremor‐dominant PD, a phenotype characterized by less motor decline and striatal degeneration, which together contribute to a more favorable clinical course (Haaxma et al. 2007). Taken together, these biological and clinical sex differences may underlie the lower burden of respiratory failure–related mortality observed in women with PD compared to men.

Previous studies have also underscored significant racial disparities in the co‐occurrence of PD and respiratory failure. White people consistently report having the greatest PD prevalence and death rates, in part because they are more likely to be diagnosed with higher baseline PD prevalence. Likewise, underdiagnosis among minorities because of lower access to healthcare and diagnostic bias could result in underreporting in earlier decades, but rising awareness over time (Wright Willis et al. 2010). Recent years have especially shown sharper increases in mortality among Hispanic and NH Black populations, which may reflect aging demographics as these populations are aging more rapidly in the United States. Since PD prevalence strongly increases with age, these groups are now showing steeper increases in PD‐related mortality (Hadidchi et al. 2025). In addition to this, the two groups have a higher risk of comorbid conditions such as diabetes, hypertension, obesity, and pulmonary disease, all of which may increase susceptibility to respiratory complications and mortality when combined with PD (Hackl et al. 2024). Lastly, minority racial and ethnic groups often face barriers to specialized neurological care access, which translates into lower utilization of advanced therapies such as deep‐brain stimulation, infusion therapies, and even optimized pharmacological management. According to a study by Aamodt et al. Black and Hispanic patients with PD were significantly less likely than White patients to receive care from neurologists or movement disorder specialists, a disparity that may contribute to worse outcomes and elevated mortality in these groups (Aamodt et al. 2023). Therefore, improvement in the division of healthcare facilities among racial groups can ensure early, comprehensive assessment and facilitate timely detection and effective treatment, which are all critical strategies for reducing the overall disease burden.

Moreover, from a geographical perspective, our study showed that the South and West carry the most significant burden in terms of absolute mortality. At the same time, the Northeast and Midwest experienced the most rapid upward trend, and the West showed the slowest rise over time. This may be attributed to the fact that both the Northeast and the Midwest regions have rapidly aging populations, with the Northeast being one of the oldest regions demographically in the United States, which increases the burden of age‐related neurodegenerative diseases, such as PD (Marras et al. 2018). The seemingly paradoxical finding of lower respiratory mortality in the Northeast despite its high prevalence of PD, particularly in states such as New York and Pennsylvania, may reflect the distinction between disease prevalence and mortality. Higher prevalence does not necessarily indicate greater mortality and may instead represent improved case ascertainment and longer survival among patients with PD. The Northeast has one of the highest densities of neurologists and movement disorder specialists in the United States, as well as greater access to multidisciplinary care and tertiary referral centers, making easier detection of disease at an early stage, referral to a specialist, as well as precise recording of death due to PD and respiratory failure (Ryu et al. 2023). Regional disparities in access to neurologists have been well‐documented in the United States, with the South and rural areas facing particularly severe shortages of movement disorder specialists. According to Willis et al. (2011), patients with PD who do not receive neurologist care are significantly more likely to experience hospitalizations, nursing home placement, and premature mortality compared to those receiving specialist care. In the South and Midwest, where neurologist density is lower, and patients often rely on general practitioners for PD management, this shortage likely contributes to worse outcomes once respiratory complications, such as aspiration pneumonia, dysphagia‐related airway obstruction, or respiratory muscle weakness, develop. Conversely, the Northeast has among the highest ratios of neurologist to patient. Although this type of access will initially lower absolute mortality through improved disease management, it also enhances diagnostic alertness and coding accuracy, thereby driving upward trends in apparent mortality in the long run. Thus, paradoxically, greater specialist availability in regions like the Northeast may simultaneously improve patient outcomes yet reveal sharper rises in reported mortality through improved surveillance and reporting.

Between 1999 and 2020, PD and respiratory failure‐related deaths were consistently higher in metropolitan areas compared to non‐metropolitan areas. This result is consistent with general population distributions, as urban areas have larger populations and higher diagnostic and reporting capacities, potentially leading to higher absolute death numbers. Nonetheless, despite these absolute mortality differences, the non‐metropolitan regions displayed significantly higher AAPCs and AAMRs compared to the metropolitan areas, with a steep inflection point from 2015 onward. There are a few potential explanations for this trend. First, non‐metropolitan regions suffer disproportionately from shortages of movement disorder specialists and specialized treatment centers, resulting in late diagnosis of PD, sub‐optimally managed motor and non‐motor comorbidities, as well as sub‐optimally prevented respiratory sequelae like aspiration pneumonia and nighttime hypoventilation (Cyr et al. 2019; McGinley et al. 2024). Second, rural populations face disadvantages in accessing healthcare, such as transportation obstacles, increased distances, and limited access to pulmonary rehabilitation or sophisticated respiratory support (Syed et al. 2013). Third, socioeconomic inequities, for example, increased smoking prevalence, chronic obstructive pulmonary disease prevalence, as well as restricted health plan coverage, might heighten susceptibility to respiratory failure among PD populations in these regions (Lowe et al. 2018). Conversely, metropolitan areas, despite their increased baseline mortality due to elevated population density and increased case ascertainment, displayed comparatively reduced rates of increase, potentially due to increased access to movement disorder medicine physicians, multidisciplinary therapies, and sophisticated respiratory therapies. The post‐2015 acceleration of mortality in both groups suggests systemic drivers, such as aging PD cohorts, rising comorbid burden, and improved documentation, that disproportionately impacted rural areas with fewer healthcare resources.

### 4.1 Limitations

This study has several significant limitations. First, the analysis relied on death certificate data, which may be subject to misclassification or underreporting of both PD and respiratory failure as causes of death. Differences in reporting practice among physicians and regional variations in coding practices may have affected observed mortality trends. Second, while the use of multiple cause‐of‐death coding is more sensitive, it cannot tell us about the actual causal sequence between PD and respiratory failure, or whether respiratory failure was necessarily a direct result of PD rather than underlying coexisting comorbidities. Lastly, data on disease duration, severity of PD, treatment exposure, and presence of important risk factors (e.g., dysphagia, pulmonary disease, or cardiovascular comorbidities) were not available, precluding adjustment for clinical heterogeneity.

Future research should focus on unraveling the complex interplay between PD progression, comorbid conditions, and respiratory complications through longitudinal clinical cohorts with detailed clinical data. Prospective trials with objective measurements of swallowing function, pulmonary mechanics, and autonomic dysfunction would help elucidate the mechanisms of respiratory failure in PD. Ultimately, research into geographic and rural–urban differences may inform targeted public health initiatives aimed at addressing disparities in care and outcomes.

## 5 Conclusion

This study shows a substantial increase in PD and respiratory failure‐related deaths in the United States between 1999 and 2020, with significant disparities by sex, race/ethnicity, geography, and urbanization. Males had significantly higher mortality than women. NH Whites had the highest increase among other racial populations. At the regional level, the Midwest and Northeast regions experienced high spikes, consistent with changes in access to health services, specialist supply, and disease prevalence. Whereas the metropolitan areas recorded most deaths, the non‐metropolitan regions recorded a late and exaggerated increase in mortality, which points to the vulnerability of rural populations with less access to specialist health services. These data illustrate growing inequities between neurological and respiratory outcomes and accentuate the importance of targeted health policy, resource distribution, and multi‐disciplinary interventions to mitigate avoidable mortality in PD. Additional research on the root causes of these differences, such as access to healthcare, socioeconomic status, and comorbidities, remains essential.

## Author Contributions

**Maryam Saghir**: validation, visualization, conceptualization, investigation, supervision, data curation, project administration, Writing – review and editing. **Amna Amir Jalal**: writing – review and editing, writing – original draft. **Eshal Saghir**: writing – original draft, writing – review and editing. **Kashmala Rahl**: writing – review and editing, writing – original draft. **Nida Shoaib**: writing – original draft, writing – review and editing. **Muhammad Affan**: writing – review and editing, writing – original draft. **Mahtab Zafar**: writing – original draft, writing – review and editing. **Nara Miriam Michaelson**: formal analysis. **Kamil Ahmad Kamil**: formal analysis.

## Funding

The authors have nothing to report.

## Ethics Statement

According to national regulations (http://nbcpakistan.org.pk/nbc‐r.html), ethics approval was not required for this study, as it did not involve any human or animal subjects. Similarly, informed consent was not necessary, since the study used publicly available, de‐identified data. As a result, institutional review board approval was not needed. We have followed the ethical standards laid out in the Declaration of Helsinki.

## Conflicts of Interest

The authors have no relevant financial or non‐financial interests to disclose.

## Supporting information




**Supplementary Tables**: brb371756‐sup‐0001‐SuppMat.docx

## Data Availability

Data is provided within the manuscript or supplementary information files.
