# Supplementary material for: Parkinson's Disease and Respiratory Failure: Epidemiologic Insights of Two Decades From the CDC‐WONDER Database
Source: Brain Behav. 2026 Sep 23;16(9):e71756. doi: 10.1002/brb3.71756 (PMC13601759; doi:10.1002/brb3.71756)
Supplement: Supplementary file 1 [file BRB3-16-e71756-s001.docx]

**Supplemental Table 1** PD and RF–related Deaths, Stratified by Sex and Race, in older Adults in the United States, 1999 to 2020.

| Year | Overall | Male | Female | NH White | NH Black or African American | NH Asian or Pacific Islander | Hispanic or Latino |
| --- | --- | --- | --- | --- | --- | --- | --- |
| 1999 | 1,886 | 1,160 | 726 | 1,762 | 73 | 47 | 69 |
| 2000 | 1,881 | 1,138 | 743 | 1,746 | 84 | 42 | 74 |
| 2001 | 1,963 | 1,181 | 782 | 1,837 | 68 | 51 | 91 |
| 2002 | 1,965 | 1,207 | 758 | 1,844 | 73 | 38 | 95 |
| 2003 | 1,988 | 1,197 | 791 | 1,848 | 75 | 56 | 80 |
| 2004 | 2,040 | 1,246 | 794 | 1,886 | 87 | 61 | 86 |
| 2005 | 2,147 | 1,263 | 884 | 1,985 | 101 | 51 | 95 |
| 2006 | 2,137 | 1,309 | 828 | 1,990 | 75 | 67 | 106 |
| 2007 | 2,195 | 1,310 | 885 | 2,025 | 94 | 70 | 129 |
| 2008 | 2,257 | 1,427 | 830 | 2,085 | 102 | 61 | 125 |
| 2009 | 2,192 | 1,370 | 822 | 2,034 | 82 | 71 | 133 |
| 2010 | 2,396 | 1,487 | 909 | 2,216 | 102 | 70 | 141 |
| 2011 | 2,487 | 1,550 | 937 | 2,279 | 105 | 94 | 143 |
| 2012 | 2,427 | 1,549 | 878 | 2,241 | 106 | 68 | 158 |
| 2013 | 2,776 | 1,776 | 1,000 | 2,521 | 150 | 97 | 181 |
| 2014 | 2,710 | 1,730 | 980 | 2,477 | 128 | 96 | 191 |
| 2015 | 3,039 | 1,957 | 1,082 | 2,783 | 123 | 122 | 213 |
| 2016 | 3,289 | 2,101 | 1,188 | 2,979 | 167 | 133 | 256 |
| 2017 | 3,639 | 2,386 | 1,253 | 3,324 | 165 | 133 | 298 |
| 2018 | 3,947 | 2,546 | 1,401 | 3,598 | 183 | 144 | 320 |
| 2019 | 3,904 | 2,577 | 1,327 | 3,495 | 205 | 192 | 288 |
| 2020 | 5,903 | 3,831 | 2,072 | 5,329 | 323 | 219 | 525 |

NH = non-Hispanic.

**Supplemental Table 2** PD and RF–related Mortality, Stratified by Place of Death in older Adults in the United States, 1999 to 2020.

| Place of Death | Deaths |
| --- | --- |
| Medical Facility - Inpatient | 24,859 |
| Medical Facility - Outpatient or ER | 1,336 |
| Medical Facility - Dead on Arrival | 80 |
| Medical Facility - Status unknown | 41 |
| Decedent's home | 9,832 |
| Hospice facility | 3,223 |
| Nursing home/long term care | 17,668 |
| Other | 1,991 |
| Place of death unknown | 138 |
| Total | 59,168 |

**Supplemental Table 3** PD and RF–related Age-Adjusted Mortality Rates per 100,000 in Adults in the United States, 1999 to 2020.

|  |  | Overall |  |  |  | Female |  |  |  | Male |  |
| --- | --- | --- | --- | --- | --- | --- | --- | --- | --- | --- | --- |
| Year | **Age Adjusted Rate** | **Age Adjusted Rate Lower 95% Confidence Interval** | **Age Adjusted Rate Upper 95% Confidence Interval** |  | **Age Adjusted Rate** | **Age Adjusted Rate Lower 95% Confidence Interval** | **Age Adjusted Rate Upper 95% Confidence Interval** |  | **Age Adjusted Rate** | **Age Adjusted Rate Lower 95% Confidence Interval** | **Age Adjusted Rate Upper 95% Confidence Interval** |
| 1999 | 5.5 | 5.26 | 5.75 |  | 3.33 | 3.09 | 3.57 |  | 9.2 | 8.67 | 9.74 |
| 2000 | 5.44 | 5.19 | 5.68 |  | 3.4 | 3.16 | 3.65 |  | 9.03 | 8.5 | 9.56 |
| 2001 | 5.53 | 5.28 | 5.77 |  | 3.5 | 3.26 | 3.75 |  | 9.16 | 8.63 | 9.69 |
| 2002 | 5.51 | 5.26 | 5.75 |  | 3.39 | 3.15 | 3.63 |  | 9.27 | 8.74 | 9.8 |
| 2003 | 5.47 | 5.23 | 5.71 |  | 3.53 | 3.28 | 3.77 |  | 8.87 | 8.36 | 9.38 |
| 2004 | 5.56 | 5.32 | 5.8 |  | 3.47 | 3.23 | 3.71 |  | 9.04 | 8.53 | 9.55 |
| 2005 | 5.8 | 5.55 | 6.04 |  | 3.81 | 3.56 | 4.07 |  | 8.98 | 8.48 | 9.47 |
| 2006 | 5.64 | 5.4 | 5.88 |  | 3.54 | 3.29 | 3.78 |  | 9.13 | 8.63 | 9.63 |
| 2007 | 5.75 | 5.51 | 5.99 |  | 3.75 | 3.5 | 4 |  | 8.95 | 8.46 | 9.43 |
| 2008 | 5.78 | 5.54 | 6.02 |  | 3.43 | 3.19 | 3.66 |  | 9.53 | 9.03 | 10.02 |
| 2009 | 5.48 | 5.25 | 5.71 |  | 3.34 | 3.11 | 3.57 |  | 8.94 | 8.46 | 9.42 |
| 2010 | 5.96 | 5.72 | 6.2 |  | 3.65 | 3.41 | 3.89 |  | 9.51 | 9.03 | 10 |
| 2011 | 6.04 | 5.8 | 6.28 |  | 3.72 | 3.48 | 3.97 |  | 9.59 | 9.12 | 10.07 |
| 2012 | 5.71 | 5.48 | 5.94 |  | 3.37 | 3.14 | 3.6 |  | 9.23 | 8.77 | 9.7 |
| 2013 | 6.37 | 6.13 | 6.61 |  | 3.82 | 3.58 | 4.06 |  | 10.3 | 9.85 | 10.81 |
| 2014 | 6.11 | 5.88 | 6.34 |  | 3.65 | 3.42 | 3.88 |  | 9.74 | 9.28 | 10.2 |
| 2015 | 6.71 | 6.47 | 6.95 |  | 4 | 3.76 | 4.24 |  | 10.7 | 10.25 | 11.21 |
| 2016 | 7.09 | 6.85 | 7.34 |  | 4.3 | 4.05 | 4.55 |  | 11.1 | 10.62 | 11.58 |
| 2017 | 7.65 | 7.4 | 7.9 |  | 4.48 | 4.23 | 4.73 |  | 12.3 | 11.77 | 12.76 |
| 2018 | 8.01 | 7.76 | 8.27 |  | 4.8 | 4.54 | 5.05 |  | 12.6 | 12.12 | 13.11 |
| 2019 | 7.7 | 7.45 | 7.94 |  | 4.43 | 4.19 | 4.67 |  | 12.3 | 11.86 | 12.82 |
| 2020 | 11.38 | 11.08 | 11.67 |  | 6.88 | 6.58 | 7.18 |  | 17.9 | 17.29 | 18.43 |

**Supplemental Table 4** PD and RF–related Age-Adjusted Mortality Rates per 100,000, Stratified by older Race in Adults in the United States, 1999 to 2020.

| Race | Year | Age Adjusted Rate | Age Adjusted Rate Lower 95% Confidence Interval | Age Adjusted Rate Upper 95% Confidence Interval |
| --- | --- | --- | --- | --- |
| Asian or Pacific Islander | 1999 | 7.15 | 5.21 | 9.56 |
| Asian or Pacific Islander | 2000 | 6.04 | 4.33 | 8.19 |
| Asian or Pacific Islander | 2001 | 6.62 | 4.9 | 8.76 |
| Asian or Pacific Islander | 2002 | 4.5 | 3.15 | 6.22 |
| Asian or Pacific Islander | 2003 | 6.26 | 4.7 | 8.17 |
| Asian or Pacific Islander | 2004 | 6.67 | 5.09 | 8.59 |
| Asian or Pacific Islander | 2005 | 5.19 | 3.85 | 6.84 |
| Asian or Pacific Islander | 2006 | 6.52 | 5.04 | 8.3 |
| Asian or Pacific Islander | 2007 | 6.38 | 4.96 | 8.07 |
| Asian or Pacific Islander | 2008 | 5.21 | 3.97 | 6.7 |
| Asian or Pacific Islander | 2009 | 5.73 | 4.47 | 7.24 |
| Asian or Pacific Islander | 2010 | 5.36 | 4.17 | 6.78 |
| Asian or Pacific Islander | 2011 | 6.66 | 5.38 | 8.16 |
| Asian or Pacific Islander | 2012 | 4.46 | 3.45 | 5.66 |
| Asian or Pacific Islander | 2013 | 6.03 | 4.88 | 7.36 |
| Asian or Pacific Islander | 2014 | 5.37 | 4.35 | 6.57 |
| Asian or Pacific Islander | 2015 | 6.38 | 5.24 | 7.52 |
| Asian or Pacific Islander | 2016 | 6.64 | 5.5 | 7.77 |
| Asian or Pacific Islander | 2017 | 6.08 | 5.04 | 7.12 |
| Asian or Pacific Islander | 2018 | 6.37 | 5.32 | 7.41 |
| Asian or Pacific Islander | 2019 | 8.04 | 6.9 | 9.19 |
| Asian or Pacific Islander | 2020 | 8.44 | 7.31 | 9.57 |
| Black or African American | 1999 | 2.73 | 2.14 | 3.43 |
| Black or African American | 2000 | 3.09 | 2.46 | 3.83 |
| Black or African American | 2001 | 2.54 | 1.97 | 3.22 |
| Black or African American | 2002 | 2.63 | 2.05 | 3.31 |
| Black or African American | 2003 | 2.69 | 2.12 | 3.38 |
| Black or African American | 2004 | 3.02 | 2.42 | 3.73 |
| Black or African American | 2005 | 3.48 | 2.8 | 4.16 |
| Black or African American | 2006 | 2.44 | 1.92 | 3.07 |
| Black or African American | 2007 | 3.14 | 2.53 | 3.84 |
| Black or African American | 2008 | 3.25 | 2.62 | 3.89 |
| Black or African American | 2009 | 2.57 | 2.04 | 3.2 |
| Black or African American | 2010 | 3.18 | 2.56 | 3.8 |
| Black or African American | 2011 | 3.1 | 2.5 | 3.69 |
| Black or African American | 2012 | 2.99 | 2.42 | 3.57 |
| Black or African American | 2013 | 4.16 | 3.49 | 4.83 |
| Black or African American | 2014 | 3.34 | 2.76 | 3.92 |
| Black or African American | 2015 | 3.14 | 2.58 | 3.71 |
| Black or African American | 2016 | 4.09 | 3.46 | 4.72 |
| Black or African American | 2017 | 3.89 | 3.29 | 4.49 |
| Black or African American | 2018 | 4.15 | 3.54 | 4.76 |
| Black or African American | 2019 | 4.34 | 3.73 | 4.94 |
| Black or African American | 2020 | 6.79 | 6.04 | 7.54 |
| White | 1999 | 5.66 | 5.4 | 5.92 |
| White | 2000 | 5.57 | 5.31 | 5.83 |
| White | 2001 | 5.77 | 5.51 | 6.04 |
| White | 2002 | 5.78 | 5.51 | 6.04 |
| White | 2003 | 5.71 | 5.45 | 5.97 |
| White | 2004 | 5.75 | 5.49 | 6.01 |
| White | 2005 | 5.96 | 5.7 | 6.23 |
| White | 2006 | 5.93 | 5.67 | 6.19 |
| White | 2007 | 5.94 | 5.68 | 6.19 |
| White | 2008 | 6.02 | 5.76 | 6.28 |
| White | 2009 | 5.76 | 5.51 | 6.01 |
| White | 2010 | 6.24 | 5.98 | 6.5 |
| White | 2011 | 6.28 | 6.02 | 6.54 |
| White | 2012 | 6.03 | 5.78 | 6.28 |
| White | 2013 | 6.67 | 6.41 | 6.94 |
| White | 2014 | 6.41 | 6.16 | 6.67 |
| White | 2015 | 7.11 | 6.84 | 7.37 |
| White | 2016 | 7.46 | 7.19 | 7.73 |
| White | 2017 | 8.11 | 7.83 | 8.39 |
| White | 2018 | 8.53 | 8.25 | 8.81 |
| White | 2019 | 8.09 | 7.82 | 8.36 |
| White | 2020 | 12.08 | 11.75 | 12.41 |
| Hispanic or Latino | 1999 | 4.94 | 3.83 | 6.27 |
| Hispanic or Latino | 2000 | 5.32 | 4.16 | 6.7 |
| Hispanic or Latino | 2001 | 5.86 | 4.7 | 7.21 |
| Hispanic or Latino | 2002 | 5.9 | 4.76 | 7.24 |
| Hispanic or Latino | 2003 | 4.67 | 3.69 | 5.83 |
| Hispanic or Latino | 2004 | 4.8 | 3.82 | 5.95 |
| Hispanic or Latino | 2005 | 4.82 | 3.89 | 5.91 |
| Hispanic or Latino | 2006 | 5.33 | 4.3 | 6.36 |
| Hispanic or Latino | 2007 | 6.2 | 5.12 | 7.28 |
| Hispanic or Latino | 2008 | 5.7 | 4.7 | 6.71 |
| Hispanic or Latino | 2009 | 5.66 | 4.7 | 6.63 |
| Hispanic or Latino | 2010 | 5.82 | 4.85 | 6.78 |
| Hispanic or Latino | 2011 | 5.5 | 4.59 | 6.4 |
| Hispanic or Latino | 2012 | 5.59 | 4.71 | 6.46 |
| Hispanic or Latino | 2013 | 6.15 | 5.25 | 7.05 |
| Hispanic or Latino | 2014 | 5.96 | 5.11 | 6.82 |
| Hispanic or Latino | 2015 | 6.22 | 5.37 | 7.06 |
| Hispanic or Latino | 2016 | 7.2 | 6.31 | 8.08 |
| Hispanic or Latino | 2017 | 7.99 | 7.07 | 8.9 |
| Hispanic or Latino | 2018 | 8.13 | 7.23 | 9.03 |
| Hispanic or Latino | 2019 | 7 | 6.18 | 7.81 |
| Hispanic or Latino | 2020 | 12.12 | 11.08 | 13.17 |

NH = non-Hispanic.

**Supplemental Table 5** PD and RF–related Age-Adjusted Mortality Rates per 100,000, Stratified by States in older Adults in the United States, 1999 to 2020.

| State | Age Adjusted Rate | Age Adjusted Rate Lower 95% Confidence Interval | Age Adjusted Rate Upper 95% Confidence Interval |
| --- | --- | --- | --- |
| Alabama | 4.59 | 4.24 | 4.95 |
| Alaska | 4 | 2.83 | 5.49 |
| Arizona | 7.27 | 6.89 | 7.65 |
| Arkansas | 9.13 | 8.51 | 9.75 |
| California | 10.57 | 10.37 | 10.78 |
| Colorado | 6.68 | 6.22 | 7.15 |
| Connecticut | 10.1 | 9.53 | 10.67 |
| Delaware | 3.58 | 2.91 | 4.36 |
| District of Columbia | 4.5 | 3.51 | 5.69 |
| Florida | 5.54 | 5.37 | 5.71 |
| Georgia | 7.37 | 7 | 7.73 |
| Hawaii | 7.05 | 6.27 | 7.82 |
| Idaho | 5.94 | 5.2 | 6.68 |
| Illinois | 4.24 | 4.03 | 4.45 |
| Indiana | 6.88 | 6.51 | 7.26 |
| Iowa | 5.55 | 5.1 | 5.99 |
| Kansas | 6.72 | 6.18 | 7.26 |
| Kentucky | 6.05 | 5.62 | 6.48 |
| Louisiana | 3.69 | 3.35 | 4.03 |
| Maine | 5.3 | 4.65 | 5.96 |
| Maryland | 3.8 | 3.5 | 4.11 |
| Massachusetts | 7.02 | 6.66 | 7.37 |
| Michigan | 3.85 | 3.63 | 4.07 |
| Minnesota | 3.75 | 3.45 | 4.05 |
| Mississippi | 9.38 | 8.72 | 10.05 |
| Missouri | 5.93 | 5.59 | 6.28 |
| Montana | 5.48 | 4.67 | 6.28 |
| Nebraska | 11.54 | 10.67 | 12.41 |
| Nevada | 5.78 | 5.19 | 6.38 |
| New Hampshire | 5.94 | 5.18 | 6.7 |
| New Jersey | 6.75 | 6.44 | 7.05 |
| New Mexico | 4 | 3.48 | 4.52 |
| New York | 6.58 | 6.37 | 6.78 |
| North Carolina | 4.42 | 4.17 | 4.68 |
| North Dakota | 6.67 | 5.62 | 7.73 |
| Ohio | 7.19 | 6.92 | 7.47 |
| Oklahoma | 5.71 | 5.26 | 6.16 |
| Oregon | 4.04 | 3.67 | 4.4 |
| Pennsylvania | 6.99 | 6.75 | 7.23 |
| Rhode Island | 6.56 | 5.73 | 7.39 |
| South Carolina | 6.36 | 5.93 | 6.8 |
| South Dakota | 5.93 | 5.02 | 6.84 |
| Tennessee | 5.95 | 5.59 | 6.3 |
| Texas | 7.23 | 7.01 | 7.46 |
| Utah | 6.86 | 6.16 | 7.56 |
| Vermont | 5.01 | 4.03 | 6 |
| Virginia | 4.98 | 4.68 | 5.28 |
| Washington | 6.65 | 6.28 | 7.02 |
| West Virginia | 6.27 | 5.66 | 6.88 |
| Wisconsin | 3.1 | 2.84 | 3.36 |
| Wyoming | 4.25 | 3.28 | 5.43 |

**Supplemental Table 6** PD and RF–related Age-Adjusted Mortality Rates per 100,000, Stratified by Census Region in older Adults in the United States, 1999 to 2020.

| Census Region | Year | Age Adjusted Rate | Age Adjusted Rate Lower 95% Confidence Interval | Age Adjusted Rate Upper 95% Confidence Interval |
| --- | --- | --- | --- | --- |
| Northeast | 1999 | 5.44 | 4.91 | 5.97 |
| Northeast | 2000 | 5.1 | 4.59 | 5.61 |
| Northeast | 2001 | 5.93 | 5.38 | 6.47 |
| Northeast | 2002 | 5.86 | 5.32 | 6.4 |
| Northeast | 2003 | 6.09 | 5.54 | 6.63 |
| Northeast | 2004 | 5.98 | 5.44 | 6.53 |
| Northeast | 2005 | 6.06 | 5.51 | 6.61 |
| Northeast | 2006 | 6.57 | 6 | 7.13 |
| Northeast | 2007 | 6.17 | 5.62 | 6.72 |
| Northeast | 2008 | 6.4 | 5.85 | 6.96 |
| Northeast | 2009 | 6.17 | 5.63 | 6.71 |
| Northeast | 2010 | 6.37 | 5.82 | 6.92 |
| Northeast | 2011 | 6.8 | 6.23 | 7.36 |
| Northeast | 2012 | 5.84 | 5.32 | 6.37 |
| Northeast | 2013 | 7.01 | 6.45 | 7.58 |
| Northeast | 2014 | 6.61 | 6.06 | 7.16 |
| Northeast | 2015 | 7.19 | 6.62 | 7.76 |
| Northeast | 2016 | 7.35 | 6.77 | 7.92 |
| Northeast | 2017 | 7.96 | 7.38 | 8.55 |
| Northeast | 2018 | 8.47 | 7.87 | 9.06 |
| Northeast | 2019 | 7.9 | 7.33 | 8.47 |
| Northeast | 2020 | 13.18 | 12.45 | 13.91 |
| Midwest | 1999 | 4.31 | 3.86 | 4.76 |
| Midwest | 2000 | 4.34 | 3.9 | 4.79 |
| Midwest | 2001 | 4.32 | 3.88 | 4.77 |
| Midwest | 2002 | 4.5 | 4.05 | 4.95 |
| Midwest | 2003 | 4.07 | 3.64 | 4.49 |
| Midwest | 2004 | 4.66 | 4.2 | 5.12 |
| Midwest | 2005 | 4.95 | 4.49 | 5.42 |
| Midwest | 2006 | 4.7 | 4.25 | 5.15 |
| Midwest | 2007 | 4.3 | 3.87 | 4.73 |
| Midwest | 2008 | 4.73 | 4.28 | 5.18 |
| Midwest | 2009 | 4.46 | 4.02 | 4.89 |
| Midwest | 2010 | 4.64 | 4.2 | 5.08 |
| Midwest | 2011 | 4.74 | 4.3 | 5.19 |
| Midwest | 2012 | 4.54 | 4.11 | 4.97 |
| Midwest | 2013 | 5.56 | 5.09 | 6.04 |
| Midwest | 2014 | 5.54 | 5.07 | 6.01 |
| Midwest | 2015 | 5 | 4.56 | 5.44 |
| Midwest | 2016 | 6.26 | 5.76 | 6.75 |
| Midwest | 2017 | 6.39 | 5.9 | 6.89 |
| Midwest | 2018 | 7.01 | 6.51 | 7.52 |
| Midwest | 2019 | 6.8 | 6.31 | 7.3 |
| Midwest | 2020 | 10.64 | 10.03 | 11.26 |
| South | 1999 | 4.78 | 4.39 | 5.18 |
| South | 2000 | 5.05 | 4.65 | 5.45 |
| South | 2001 | 4.98 | 4.58 | 5.37 |
| South | 2002 | 4.8 | 4.42 | 5.19 |
| South | 2003 | 4.81 | 4.43 | 5.2 |
| South | 2004 | 4.97 | 4.59 | 5.36 |
| South | 2005 | 5.11 | 4.72 | 5.5 |
| South | 2006 | 4.5 | 4.14 | 4.86 |
| South | 2007 | 5.17 | 4.79 | 5.56 |
| South | 2008 | 5.12 | 4.74 | 5.49 |
| South | 2009 | 4.69 | 4.33 | 5.05 |
| South | 2010 | 5.62 | 5.23 | 6.01 |
| South | 2011 | 5.3 | 4.93 | 5.67 |
| South | 2012 | 4.9 | 4.55 | 5.25 |
| South | 2013 | 6.11 | 5.72 | 6.5 |
| South | 2014 | 5.51 | 5.15 | 5.88 |
| South | 2015 | 6.38 | 6 | 6.77 |
| South | 2016 | 6.19 | 5.82 | 6.57 |
| South | 2017 | 7.19 | 6.79 | 7.59 |
| South | 2018 | 7.55 | 7.15 | 7.95 |
| South | 2019 | 7.17 | 6.79 | 7.56 |
| South | 2020 | 10.1 | 9.66 | 10.55 |
| West | 1999 | 8.24 | 7.55 | 8.93 |
| West | 2000 | 7.8 | 7.13 | 8.46 |
| West | 2001 | 7.74 | 7.09 | 8.4 |
| West | 2002 | 7.55 | 6.91 | 8.19 |
| West | 2003 | 7.67 | 7.03 | 8.31 |
| West | 2004 | 7.39 | 6.76 | 8.01 |
| West | 2005 | 7.6 | 6.98 | 8.22 |
| West | 2006 | 7.81 | 7.18 | 8.43 |
| West | 2007 | 7.88 | 7.26 | 8.5 |
| West | 2008 | 7.41 | 6.82 | 8 |
| West | 2009 | 7.47 | 6.88 | 8.06 |
| West | 2010 | 7.57 | 6.98 | 8.16 |
| West | 2011 | 7.9 | 7.31 | 8.49 |
| West | 2012 | 8.28 | 7.68 | 8.88 |
| West | 2013 | 7.21 | 6.66 | 7.76 |
| West | 2014 | 7.32 | 6.77 | 7.86 |
| West | 2015 | 8.54 | 7.96 | 9.12 |
| West | 2016 | 9.23 | 8.63 | 9.83 |
| West | 2017 | 9.29 | 8.7 | 9.88 |
| West | 2018 | 9.33 | 8.75 | 9.91 |
| West | 2019 | 9.41 | 8.83 | 9.98 |
| West | 2020 | 12.9 | 12.24 | 13.56 |

**Supplemental Table 7** PD and RF–related Age-Adjusted Mortality Rates per 100,000, Stratified by Urban-Rural Classification in older Adults in the United States, 1999 to 2020.

|  |  | Metropolitan |  |  |  | Non-metropolitan |  |
| --- | --- | --- | --- | --- | --- | --- | --- |
| Year | **Age Adjusted Rate** | **Age Adjusted Rate Lower 95% Confidence Interval** | **Age Adjusted Rate Upper 95% Confidence Interval** |  | **Age Adjusted Rate** | **Age Adjusted Rate Lower 95% Confidence Interval** | **Age Adjusted Rate Upper 95% Confidence Interval** |
| 1999 | 5.58 | 5.3 | 5.86 |  | 5.08 | 4.54 | 5.63 |
| 2000 | 5.5 | 5.22 | 5.77 |  | 4.97 | 4.44 | 5.51 |
| 2001 | 5.76 | 5.48 | 6.04 |  | 4.87 | 4.35 | 5.4 |
| 2002 | 5.75 | 5.47 | 6.03 |  | 4.39 | 3.9 | 4.89 |
| 2003 | 5.67 | 5.4 | 5.95 |  | 4.59 | 4.08 | 5.1 |
| 2004 | 5.77 | 5.5 | 6.04 |  | 4.82 | 4.3 | 5.34 |
| 2005 | 6 | 5.72 | 6.27 |  | 4.59 | 4.09 | 5.09 |
| 2006 | 5.83 | 5.56 | 6.1 |  | 4.87 | 4.36 | 5.39 |
| 2007 | 5.92 | 5.65 | 6.19 |  | 4.77 | 4.26 | 5.28 |
| 2008 | 5.91 | 5.65 | 6.18 |  | 5.04 | 4.52 | 5.56 |
| 2009 | 5.68 | 5.42 | 5.94 |  | 4.52 | 4.03 | 5.02 |
| 2010 | 6.08 | 5.82 | 6.35 |  | 5.38 | 4.84 | 5.91 |
| 2011 | 6.24 | 5.97 | 6.51 |  | 5.13 | 4.62 | 5.65 |
| 2012 | 5.85 | 5.59 | 6.11 |  | 5.33 | 4.81 | 5.86 |
| 2013 | 6.68 | 6.41 | 6.95 |  | 5.01 | 4.51 | 5.52 |
| 2014 | 6.23 | 5.98 | 6.49 |  | 5.5 | 4.97 | 6.03 |
| 2015 | 6.89 | 6.62 | 7.16 |  | 5.79 | 5.25 | 6.32 |
| 2016 | 7.21 | 6.94 | 7.48 |  | 6.46 | 5.9 | 7.02 |
| 2017 | 7.72 | 7.44 | 7.99 |  | 7.12 | 6.54 | 7.7 |
| 2018 | 8.03 | 7.76 | 8.31 |  | 7.81 | 7.21 | 8.41 |
| 2019 | 7.82 | 7.55 | 8.09 |  | 7.38 | 6.81 | 7.96 |
| 2020 | 11.21 | 10.89 | 11.52 |  | 12.3 | 11.56 | 13.04 |
